# Monitoring Antibiotic Use and Residue in Freshwater Aquaculture for Domestic Use in Vietnam

**DOI:** 10.1007/s10393-014-1006-z

**Published:** 2015-01-06

**Authors:** Dang Kim Pham, Jacqueline Chu, Nga Thuy Do, François Brose, Guy Degand, Philippe Delahaut, Edwin De Pauw, Caroline Douny, Kinh Van Nguyen, Ton Dinh Vu, Marie-Louise Scippo, Heiman F. L. Wertheim

**Affiliations:** 1 Faculty of Animal Science and Aquaculture (FASA), Hanoi University of Agriculture, Hanoi, Vietnam; 2 Princeton University, Princeton, NJ USA; 3 Wellcome Trust Major Overseas Program, Oxford University Clinical Research Unit, Hanoi, Vietnam; 4 Global Antibiotic Resistance Partnership, Hanoi, Vietnam; 5 Laboratory of Food Analysis, Department of Food Sciences, CART (Centre of Analytical Research & Technology), University of Liège, Liège, Belgium; 6 Laboratory of Hormonology, CER Groupe, Marloie, Belgium; 7 Laboratory of Mass Spectrometry, Department of Chemistry, CART (Centre of Analytical Research & Technology), University of Liège, Liège, Belgium; 8 National Hospital for Tropical Diseases, Hanoi, Vietnam; 9 Centre for Tropical Medicine, Nuffield Department of Clinical Medicine, Oxford, UK

**Keywords:** antibiotics, resistance, aquaculture, vietnam, residue

## Abstract

Vietnam is an important producer of aquaculture products, and aquatic products are essential to the Vietnamese diet. However, Vietnam also has very little enforced regulation pertaining to antibiotic usage in domestic aquaculture, which raises concerns for antibiotic resistance in pathogenic bacteria. In this study, analysis was conducted on the presence of antibiotic residues in domestically sold fish and shrimp raised in freshwater farms in Vietnam, and an assessment of farmers’ knowledge of proper antibiotics usage was performed. The results indicated that a quarter of tested aquaculture products were antibiotic screening test positive, and there is a general lack of knowledge about the purpose and proper usage of antibiotics by aquaculture producers. Farmers’ decision-making processes about antimicrobial use are influenced by biased sources of information, such as drug manufacturers and sellers, and by financial incentives.

**Electronic supplementary material:**

The online version of this article (doi:10.1007/s10393-014-1006-z) contains supplementary material, which is available to authorized users.

## Introduction

Aquaculture production is an important source of pollution of veterinary medicines into the environment (Pruden et al. 2013; Rico and Van den Brink 2014). About 90% of the global aquaculture production is produced in Asia (Thuy et al. 2011). According to 2009 statistics from the Food and Agriculture Organization of the United Nations (FAO), Vietnam has become the third largest producer of aquaculture products, behind China and India (Anonymous 2009). As of 2010, there are 37,142 fish farms in Vietnam, which produce over 2,700 thousand tons of aquatic products per year (Anonymous 2010b). While a large amount of Vietnam’s aquatic products are exported, the demand in domestic markets is also high as aquatic products are an essential part of the Vietnamese diet. Results of a global survey on antimicrobial use in aquaculture showed high use of antibiotics in fish and shrimp farming, including prohibited drugs (Tusevljak et al. 2013), and this raised critical human health concerns. Therefore, understanding the processes through which aquatic animals, such as finfish and shrimp, are raised and eventually brought to the dinner table is of extreme importance.

A large number of aquaculture farms in Vietnam are highly intensive in order to increase the yield per unit area. In a study on intensive shrimp farming in the Mekong Delta, many farmers are attracted to the initially high yields and profits brought by intensive farming (Lan 2013). However, these yields decrease and the profits disappear in less than a decade (Nguyen and Ford 2010). Thus, intensive shrimp farming is not as profitable or sustainable as farmers would expect. An important factor that contributes to this drop in yield is disease outbreaks, which in turn are exasperated by the high stock density of intensive farming (Subasinghe et al. 2001; Nguyen and Ford 2010). In attempt to salvage their farms and improve yields, farmers resort to using a variety of antibiotics. A study conducted on antibiotic contamination in northern Vietnam indicates the presence of three major antibiotic classes in aquatic environments: sulfonamides, diaminopyrimidine (trimethoprim), and macrolides (Hoa et al. 2011). Research by Le and Munekage on shrimp farms in mangrove areas in Vietnam indicates the presence of similar antibiotics in both northern and southern Vietnam (Le et al. 2005). In addition to sulfonamides and trimethoprim, they also detected quinolones: norfloxacin and oxolinic acid (Le et al. 2005).

The use of antibiotics in aquaculture systems can create serious economic and health problems. Antibiotic residues have been found in several aquatic products from Vietnam and other Asian countries (Canada-Canada et al. 2009; Won et al. 2011; He et al. 2012). Because of stringent regulations from the U.S. and European Union (EU), the issue of antibiotic residues in aquatic products for export has been mostly resolved; however, there are no such well-enforced regulations for Vietnam’s domestic markets. Since fish and other aquatic products represent a very large portion of their diet, Vietnamese people are potentially being exposed on a daily basis to antibiotic residues, which can even in sub-therapeutic concentrations lead to an increase in antibiotic resistance (Van Anrooy 2003; Pruden et al. 2013).

The main danger of antibiotic use is the development and selection of antibiotic resistant pathogens. Since many of the antibiotics used are non-biodegradable, industrial antibiotics used in aquaculture farms can place intense selective pressure on aquatic microbial populations. The presence of a large number of antibiotic resistance genes in these populations is evidence of this selective pressure (Zhang et al. 2009; Pruden et al. 2013). The situation in Vietnam is amplified by the integrated agriculture-aquaculture (IAA) farming system encouraged by the government, which often involves an aquaculture system that is sustained through the addition of human and livestock waste. This creates an environment that greatly increases the ease through which antibiotic resistance genes present in livestock or humans are transferred to aquaculture and from there into waterbodies that is used by humans and animals for consumption (Hoa et al. 2011). These antibiotic resistance genes can be easily transferred to both human and animal pathogens, creating a severe health risk by greatly limiting the antibiotics that can be used to treat infectious diseases (Zhang et al. 2009; Pruden et al. 2013).

Due to the lack of regulation, there is very little information about antibiotics used in aquaculture for the domestic markets of Vietnam ad whether these is any antibiotic residue present. This study attempts to address this issue by providing field research on antibiotic use in freshwater aquaculture as well as a laboratory analysis for antibiotic residue in fish and shrimp destined for Vietnamese domestic markets.

## Methods

We conducted two studies: (1) a cross-sectional study was conducted from July 2011 to August 2011 to determine how antimicrobials are used on aquaculture farms and to determine the knowledge of Vietnamese aquaculture farmers had on the purpose and safety of antibiotics usage in freshwater farming; (2) sampling of fish and shrimp from regional fresh markets for antibiotic residue analysis.

We estimated that ~20% of farms would not use antibiotics, which would require a sample size of 100 farms to estimate this with 5% confidence limits and a power of 80%.

### Sampling Locations

**Figure 1:**
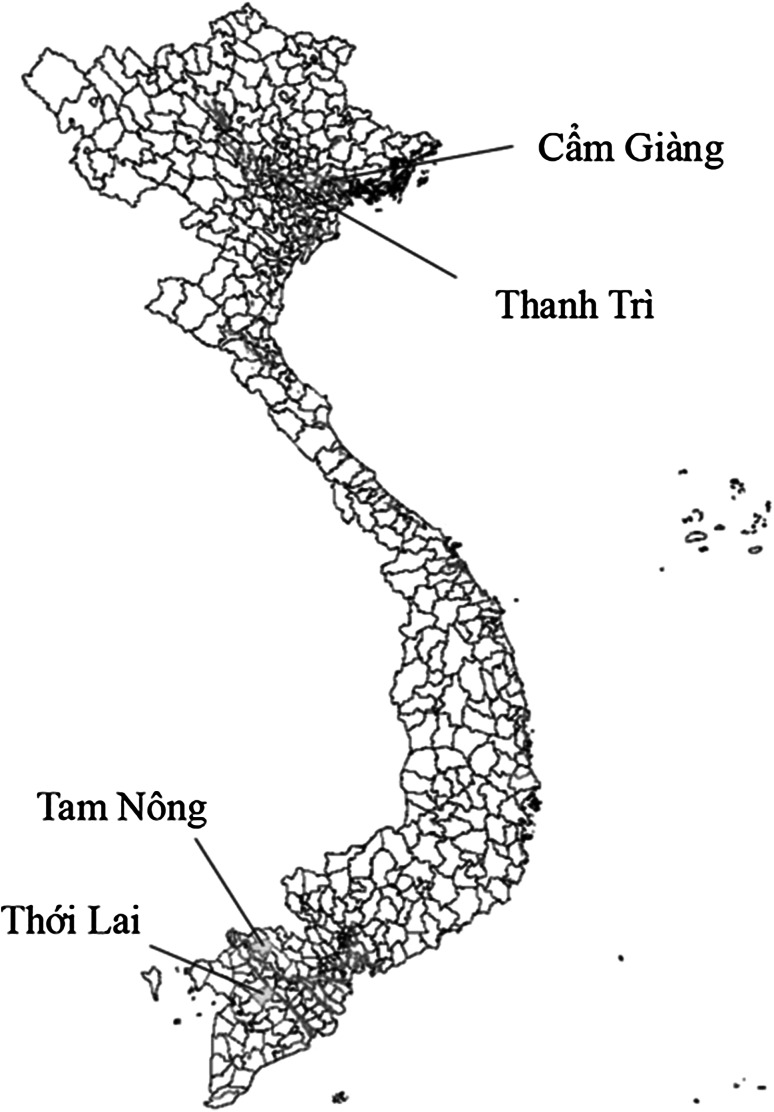
Geographical locations of the study areas.

Two regions of Vietnam were selected for study: the Mekong River Delta (MRD), in southern Vietnam, and the Red River Delta (RRD), in northern Vietnam (Fig. 1). Both areas are large producers of Vietnamese aquaculture (Worldbank 2005). From each of these regions, two smaller areas that are representative of the regions’ geography and farm size were selected. For the Mekong River Delta, Can Tho in the Thoi Lai district and Dong Thap in the Tam Nong district were chosen, and, for the Red River Delta, Hai Duong in the Cam Giang district and Hanoi in the Thanh Tri district were chosen.

### Sampling and Data Collection

#### Survey of Farmers’ Knowledge

Ninety-four farms in two study areas, the MRD and the RRD, were randomly selected using ‘randbetween’ function in Excel (Microsoft, USA) from a list of aquaculture farms (*n* = 535 in RRD and *n* = 622 in MRD) obtained from the Department of Agriculture. For the interview, the farm owners were approached, who were free to refuse to participate in the study. All data were anonymous to protect farmer’s identity by not using names or other identifiers on the forms. In case of farmer absence, the farm would be visited the next day. Field data on farmers’ knowledge of antibiotics use were collected through direct interview in the Vietnamese language with the farmers. The survey questionnaire was developed in Vietnamese language by Hanoi Agricultural University, which was peer reviewed by external experts (content validation) and piloted before use for face validation (see supplementary information). The interviews in both regions were done by the same interview team. The survey was approved by the Institutional Review Board of the Hanoi Agricultural University.

#### Antibiotic Residue Analysis

Data on antibiotic residues were obtained by purchasing a total of 50 shrimp samples and 50 fish samples (any species) from local markets in the study areas (at least 300 g per sample required). For transport to the laboratory, each sample was packaged in polyethylene and stored in an insulating container at a cool temperature. The local market sold mainly fish/shrimp from local region but the exact source is unknown. The samples reached the laboratory within 3 h. At the laboratory, the bones and offal of the samples were removed, and the rest of the sample was milled to create a homogenous paste. Each sample was then stored in sealed plastic boxes at −20°C until antibiotic residue analysis.

All samples were first analyzed using the microbiological inhibition test (New Two Plate Test or NTPT) at the laboratory of the Hanoi Agricultural University to rapidly screen for samples containing antibiotic residues, as described previously (Dang et al. 2010). These positive samples then underwent a post-screening step in order to identify the antibiotic class of the residues detected. A confirmation analysis was performed at Laboratory of Food Analysis, CART (Centre of Analytical Research & Technology), University of Liège in Belgium, to identify and quantify the molecule(s) in positive samples after the post-screening step.

(Fluoro) quinolones and tetracyclines are often used in aquaculture production (Tusevljak et al. 2013). Therefore in this study, samples which displayed a positive response after the first screening step were analyzed using two different post-screening tests, the Tetrasensor^®^ test for tetracyclines and an direct competitive enzyme-linked immunosorbent assay (ELISA) for (fluoro)quinolones. Tetrasensor^®^, provided by Unisensor, S.A. (Wandre, Liege, Belgium), is a receptor-based assay using dipsticks for rapid screening of tetracyclines in animal tissues (limit of detection 20 µg kg^−1^). The ELISA kit (CER, Marloie, Belgium) provides quantitative analysis of a broad range of (fluoro) quinolones residues in various matrices, using an anti-sarafloxacin antibody, a norfloxacin-peroxydase conjuguate, and a sarafloxacin calibration curve (EIA Fluoroquinolones 2 h E.G.3). Positive samples were analyzed by liquid chromatography coupled to mass spectrometry (LC–MS).

##### Quantification of (Fluoro) Quinolones

The sample extraction procedure was adapted from the method described in the papers of Toussaint et al. (2002; van Vyncht et al. 2002). Prior to extraction, the samples (1 g of tissue) were spiked briefly with 100 μL of lomefloxacin and 2-phenyl-4-quinoline carboxylic acid (Cinchophen), used as internal standards (both from Sigma-Aldrich) (3 μg L^−1^ in methanol). The extraction step was performed using 10 mL acetonitrile. The sample extract was evaporated to dryness and ammonium acetate buffer (5 mM, pH 4) was then added to obtain a final volume of 2 mL. The purification step was performed using SPE cartridges (SDB-RPS, 3 M Empore). Analytes were eluted with 4 mL of a mixture of methanol and ammonium hydroxide 1 M (75:25, v/v). The eluate was then evaporated to dryness and reconstituted with 300 μL of formic acid (pH 2.5) (prepared by adding formic acid 98% to 1 L of water to obtain a pH of 2.5). A calibration curve containing 13 (fluoro) quinolone standards—norfloxacin, ofloxacin, cinoxacin, flumequine, enoxacin, oxolinic acid, nalidixic acid, enrofloxacin, sarafloxacine, difloxacine, and danofloxacin mesylate from SIGMA (St Louis, MO, USA) and marbofloxacin from Vetoquinol (Belgium)–was prepared with the same procedures used for the samples, using fortified blank tissue (fish or shrimp) at five different concentrations around the EU Maximum Residue Levels (MRLs) for each antibiotic: 100 μg kg^−1^ for oxolinic acid and the sum of ciprofloxacin and enrofloxacin, 200 μg kg^−1^ for flumequine and 300 μg kg^−1^ for difloxacine (Anonymous 2010a). For antibiotics with no stated MRL in fish (sarafloxacin, norfloxacin, ofloxacin, cinoxacin, nalidixic acid, enoxacin, marbofloxacin), a reference concentration of 100 μg kg^−1^ was chosen, corresponding to the lowest MRL allowed for fluoroquinolones in the EU. Ciprofloxacin was included in that list because it is a marker residue of enrofloxacin. Blank tissues were coming from fish and shrimp bought in Belgian supermarkets. They were first checked by LC–MS to not contain any antibiotic residue.

Identification and quantification were performed by LC–MS/MS on a 2690 Alliance separation Modules integrated auto sampler, solvent delivery system, and column heater coupled to a Quattro Ultima Platinum triple-quadrupole mass spectrometer (Micromass, Manchester, UK). The mass spectrometer was equipped with an electrospray ionization (ESI) interface. The LC column used was a Polaris C18A 3 μm (150 × 2.0 mm) with a Chromsep guard column SS (10 × 2.0 mm) both from Varian. The limit of quantification (LOQ) for most quinolones is 12.5–25 μg/kg.

##### Quantification of Tetracyclines

Prior to extraction, the samples (5 g of tissue) were spiked with methacycline (Dr. Ehrenstorfer, Augsburg, Germany) used as an internal standard. Tetracyclines residues were then extracted twice with 0.05 M succinate buffer (pH 4), 20 mg of EDTA, and 15 mL of hexane to remove fat. After shaking vigorously, the samples were centrifuged; hexane was removed and the aqueous phase was transferred into a new tube. This extraction was reproduced without hexane. Both pooled supernatants were applied on a pre-conditioned SPE column (OASIS hydrophobic lipophilic-balanced (HBL), 6 mL, 200 mg, Waters Corp, Milford, MA, USA). The column was washed with 20 mL of water/methanol (95/5, v/v) and degreased with 5 mL of hexane. The tetracycline residues were eluted with 5 mL methanol. The extracts were evaporated to dryness at 45°C under a flow of nitrogen. The dry residue was dissolved in 1 mL of water/methanol (70/30, v/v).

The method used for quantification of tetracyclines (tetracycline, oxytetracycline, and their metabolites, the 4-epimer) was based on the method described by Xu et al. (2008). In short, the final separation and detection were performed by LC–MS/MS using a Quattro Ultima tandem mass spectrometer coupled to a HPLC 2690 separation module system and integrated autosampler (Micromass, Manchester, UK). The tetracyclines were detected in positive ESI in multiple reactions monitoring acquisition mode. A Sunfire C18 column (150 mm × 2.1 mm, 5.0 μm particle size) (Waters, Milford, MA, USA) was used for the chromatographic separation. The limit of quantification was 1 μg kg^−1^ for each analyte.

### Analysis

Farms were classified as intensive or non-intensive according to definition by the Food and Agricultural Organization of the United Nations (Edwards and Demaine 1998). Collected data were entered into an Excel spreadsheet and checked for quality by an independent data analyst. Data were analyzed by SPSS software, version 19 (SPSS Inc., USA) using descriptive statistics as appropriate, including: medians and interquartile ranges (IQR), means, proportions, and their 95% confidence intervals (95% CI). Parametric and non-parametric statistical testing was used as appropriate. *P* values under 0.05 were considered significant (2-sided).

## Results

### General Characteristics of Surveyed Farms

A total of 94 farms were selected and enrolled in the study; 48 from the RRD and 46 from the MRD. No farmers refused to participate. Among the selected farms, the majority raised either shrimp or fish, just 10 (11%) farms raised both. A significantly greater proportion was shrimp farms in the MRD as compared to RRD with 7/48 shrimp farms (Table 1) (24/46) (*P* < 0.05).

In fish farms, the most common types of species raised are tilapia, common carp, grass carp, snakehead fish, and catfish. Shrimp farms raise species of giant prawn. Overall, 85.1% (80/94) of farms use IAA farm system, in which aquaculture animals are raised with other farm animals, such as pigs or chickens, or raised within or alongside rice fields. A higher percentage of farms in the MRD (69.6%) are intensive as compared to the RRD (43.8%), as all shrimp farms surveyed are intensive.

The median pond surface area of the fish farms in the RRD (3,500 m^2^) was smaller than the median size of fish farms in the MRD (11,500 m^2^; Table 1). The total yield of aquaculture farms varied from 0.3 to 200 tons per year, with a median of 2.9 tons in the RRD and 3.4 tons in the MRD. The shrimp farms in the MRD had a much lower yield per year compared to shrimp farms in the RRD: 2.9 versus 8.2 tons, respectively. The yields of fish farms for both deltas were 2.5 tons in RRD and 3.9 tons in MRD. See Table 1 for more detailed descriptive data of the farms.

**Table 1:** Descriptive Data on Surveyed Fish and Shrimp Farms.

	Red River Delta		Mekong River Delta	
	Fish farm	Shrimp farm	Fish farm	Shrimp farm
	*n* = 41	*n* = 7	*n* = 22	*n* = 24
Education level of farmer based on average years in school	7.6	8.6	6.0	5.0
Average number of people living at the farm	4.1	4.7	4.5	4.8
Median number of ponds (IQR)	2 (1–3)	3 (2–5)	1 (1–2)	1.5 (1–2)
Median number of harvests per year (IQR)	1.5 (1–2)	3 (2–3)	1 (1–2)	1 (1–3.5)
Median water surface area (m^2^, IQR)	3,500	14,000	11,500	20,000
	(2,520–8,820)	(9,000–30,000)	(1,500–20725)	(10,000–34,500)
Median stocking density (individuals/m^2^, IQR)	1.1	15.0	5.9	12.3
	(0.8–2.0)	(14.0–15.0)	(0.7–32.1)	(10.0–15.2)
Median yield (tons of aquaculture harvested per year, IQR)	2.5 (1.5–5.7)	8.2 (3.4–14.4)	3.9 (1.9–23.3)	2.9 (0.8–5.0)

*IQR* interquartile range.

### Use of Antibiotics in Surveyed Farms

**Figure 2:**
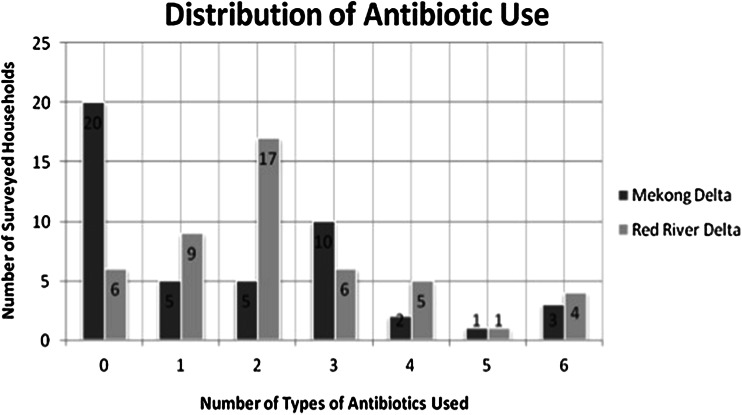
Number of types of antibiotics used by surveyed region.

There are a large number of freshwater farms that use antibiotics in raising fish or shrimp. Of all the farms surveyed (*n* = 94), 68 (72.3%) of them used at least one antibiotic at any time in the production cycle (Fig. 2). Twenty-six out of the 94 farms reported not to use antibiotics during the surveyed period, while 7 out of 94 farms used 6 types of antibiotics (Fig. 2). A total of 10 different classes of antibiotics were identified that were used by the farms. The three most commonly used antibiotics were trimethoprim (30.8%), oxytetracycline (30.9%), and sulfamethoxazole (41.5%; see Table 2). There was also considerable usage of sulfadiazine (17.0%). For 88.3% of the farms, a variety of chemicals, including lime and probiotics, were used to maintain water quality. Twelve of the antibiotics used in aquaculture (8/26, 30.8%) are on the list of the in 2011 revised World Health Organization (WHO) critically important antimicrobials for human use, particularly beta-lactam antibiotics, and quinolones (Table 2) (Anonymous 2012). Furthermore, a considerable number of farms (23.4%; 22/94) used antibiotics up to harvest time.

**Table 2:** Antibiotics Used in Freshwater Aquaculture Farms.

Class	Antibiotic	Number of farms in which antibiotic is used (*n* = 94)	On the WHO’s critically important antimicrobials list (2011)
β-lactams	Ampicillin	5	Critically important
	Penicillin	1	Critically important
	Amoxicillin	4	Critically important
	Cephalexin	2	Highly important
Aminoglycosides	Neomycin	5	Critically important
	Kanamycin	5	Critically important
Diaminopyrimidine	Trimethoprim	29	Highly important
Macrolides	Erythromycin	1	Critically important
Fenicols	Florfenicol	7	Veterinary use only
	Thiamfenicol	5	Highly important
Tetracyclines	Doxycycline	1	Highly important
	Oxytetracycline	29	Highly important
	Chlortetracycline	2	Highly important
	Tetracycline	8	Highly important
Polymyxins	Colistin	6	Critically important
Fluoroquinolones	Enrofloxacin	8	Veterinary use only
	Norfloxacin	5	Critically important
	Ofloxacin	1	Critically important
	Ciprofloxacin	1	Critically important
	Flumequine	1	Critically important
Sulfonamides	Sulfamethoxazol	39	Highly important
	Sulfadiazine/Sulfadimidine	16	Highly important
Rifamycin	Rifampicin	2	Critically important

On farms that reported using antibiotics, on average 3.3 kg (95% CI 2.4–4.3) of antibiotics are used for every ton of aquatic product. Antibiotics are generally stored in very rudimentary conditions; they are often placed in a plastic bag and hung on the wall or in a corner of the family home. No legitimate storage facilities are used since antibiotics are bought for only one treatment period of 3–4 days. Most farms administer the antibiotics by mixing it into the feed, either by hand or with a shovel, despite reduced food intake by ill fish or shrimp.

The farms that used antibiotics did have, on average, a higher density of fish or shrimp per m^2^ of water surface: 10.6 versus 7.8 (*P* = 0.064). Also the total production per year in tons per m^2^ was higher in those using antibiotics: 24.8 versus 3.5 (*P* = 0.026).

### Farmers’ Knowledge About Antibiotics and Their Decision-Making Process

Out of all the farms surveyed, only 15 out of 94 stated that they knew the regulations relating to aquaculture and antibiotic use. Also, of the 94 farms surveyed, not a single one of them uses a logbook for keeping track of antibiotics used, despite recommendation from the Vietnamese government. Instead, farmers often recorded budget or finance information regarding the cost of veterinary feed, drugs, and disinfectants.

For those farmers that did not use antibiotics, they stated that their fish or shrimp remained healthy, and, therefore, they did not need to buy antibiotics. Through a survey conducted on the farmers’ opinions on antibiotic use, it seems that farmers have limited knowledge about the purpose of antibiotics. A total of five questions were asked, and, for each question, at least a quarter of the farmers stated ‘Do not know’ as a response. Over half of the farmers surveyed agreed with the idea that antibiotics could be used for prophylaxis, including 30.9% of farmers who strongly agreed. There were 44.8% of farmers who believed that antibiotics had no effect on curing diseases. Thirty-six of the 42 farmers who held that opinion still used antibiotics for either treatment or prophylaxis, which accounts for 52.9% of the farmers who used antibiotics.

The majority of farm owners depend on consultations from drug sellers and manufacturers for antibiotics, who may encourage farm owners to use antibiotics unnecessarily in order to make a profit. Some farmers sought the advice of veterinarians (32/94) or other farmers (3/94), but often also relied on their own experience (29/94). Most farmers will go directly to a veterinary drug store to buy antibiotics; only 34.0% of farm owners will use antibiotics based on a veterinarian’s prescription. Drug and feed manufacturers also influence aquaculture farmers through seminars that are jointly run with the local government. Of the surveyed farms, 56.4% stated that they regularly attended these seminars. Excluding the seminars, the local government seems to play a minor role in the production of aquaculture. Only 31.9% of farms have been inspected by aquaculture officials, and, even then, the farms were usually tested for water quality; no thorough examinations were performed on the animals themselves. A fifth of the farms also stated participation in aquaculture or fishery clubs, a venue for farmers to share knowledge.

### Analysis of Antibiotic Residues

A total of 53 shrimps samples and 51 fish samples bought from regional markets (not from the investigated farms) were tested for antibiotic residues. Residue screening tests indicated that a total of 26.9% (28/104) of samples were detected to contain antibiotic residues (Table 3). Post-screening results indicate that, of 28 suspected samples, 7 samples (1 shrimp and 6 fish) contain (fluoro) quinolone residue; 18 samples (13 shrimp and 5 fish) contain tetracycline residue (Table 2). However, the result of quantification analysis by LC/MS show that no shrimp samples contained tetracycline residues at concentrations above the set detection threshold. These may, therefore, be Tetrasensor false positive results as this test is not validated for shrimp tissue and the test is sensitive to salt concentration (Table 4). For fish, 4 samples contained tetracycline residues at concentrations above the set detection threshold, and 6 samples were confirmed to contain fluoroquinolones residues.

**Table 3:** Antibiotic Residue Screening Results (Using the NTPT) of Collected Samples from Local Markets.

Region		Sample type		Total by region
		Fish	Shrimp	
Red River Delta	Number of analyzed samples	30	23	53
	Positive samples	8	3	11
	Percent positive	26.7%	13.0%	20.7%
Mekong Delta	Number of analyzed samples	21	30	51
	Positive samples	7	10	17
	Percent positive	33.3%	33.3%	33.3%
Total	Number of analyzed samples	51	53	104
	Positive samples	15	13	28
	Percent positive	29.4%	24.53%	26.92%

**Table 4:** Post-screening and Confirmation Results of Suspect Samples.

	Shrimp sample (*n* = 13)	Fish sample (*n* = 15)	Total (*n* = 27)
Number of positive samples with (fluoro)quinolones post-screening test (ELISA)	1	6	7
Number of confirmed samples containing (fluoro)quinolones by LC/MS	0	6	6
		3 samples: ENR (<12 ppb) and CIP (<12 ppb);	
		2 samples: ENR (13 ppb) and CIP (14 ppb);	
		1 sample: ENR (28 ppb),CIP (66 ppb) and NOR (trace)	
Number of positive samples with tetracyclines post-screening test (Tetrasensor)	13	5	18
Number of confirmed samples containing tetracyclines by LC/MS	0	4	4
		1 sample with OTC (17 ppb) and 4-epi OTC (2 ppb)	
		4-Epi OTC (4 ppb)	
		OTC (25 ppb), 4-epi OTC (5 ppb)	
		4-Epi OTC (3 ppb)	

*ENR* enrofloxacin, *CIP* ciprofloxacin, *NOR* norfloxacin, *OTC* oxytetracycline.

## Discussion

While multiple studies have been conducted on the presence of antibiotic resistance and antibiotic use in aquaculture or other farm environments (Giraud et al. 2006; Hoa et al. 2011; Tusevljak et al. 2013), there are relatively few studies that directly gather information regarding farmers’ knowledge of antibiotic usage and safety, especially in Vietnam, despite the country’s status as a major producer of aquaculture products. Even among studies conducted in Vietnam, the main focus has been on the Mekong Delta, and with main focus shrimp farming (Le et al. 2005; Thuy et al. 2011; Lan 2013). The goal of this study was to gather data regarding the use of antibiotics by freshwater aquaculture farmers in Vietnam who mainly produced for the domestic market. The results are thus not applicable to farmers producing for export.

The results of this study indicate that farmers freely use antibiotics despite their limited knowledge about antibiotics; including antibiotics on the WHO lost of critically important antimicrobials and banned antimicrobials like fluoroquinolones. Though concerning, this provides an indication of the honesty and reliability of the farmers’ survey responses since they are, for the most part, unaware of their misuse of antibiotics. Economic incentives drove their decision to use antibiotics. A relative small random sample size was used, which created problems in data analysis since there was simply inadequate data to draw conclusions pertaining to more detailed aspects of the study. However, the data collected were enough to create a general view and overall sense of fresh water shrimp and fish farmers’ antibiotics knowledge.

We found that fish and shrimp bought at a regional market were antibiotic residue screening test positive in about 25%. Fluoroquinolone and tetracycline residue were detected in fish sold on the market, indicating lack of adequate withdrawal times. Also the survey indicated that a considerable number of farms (23.4%) used antibiotics up to harvest time. These fish or shrimp were often suffering from illness; therefore antibiotics were used to maintain an appearance of health in order to minimize losses and gain back the capital. The decision to use antibiotics is a matter of economic practicality rather than health or safety. These findings are similar that those found in a previous study about chicken and pork meat produced in Vietnam (Baquero et al. 2008; Pruden et al. 2013). A limitation is that we were not able to test fish and shrimp from the farms in the study or link our findings to antibiotic resistance levels.

Diseases are rarely diagnosed; farmers will either use an antibiotic that seemed to work previously or switch antibiotics until they find one that seems to cure the disease. This may explain many farmers’ use of multiple antibiotics. There is, however, no evidence from this study that indicates that the use of multiple antibiotics is effective. The proper, recommended method of preventing or managing disease outbreaks is maintaining water quality and decreasing stress in the animals caused by overcrowding and high stocking densities (Subasinghe et al. 2001; Nguyen and Ford 2010).

The farmers’ lack of knowledge is most aptly illustrated through questions asked about their opinions on antibiotics use, which resulted in contradictions between their stated opinions and their actions. Despite stating that antibiotics seem to have no actual effect on curing disease, a large proportion of farmers with that opinion still used antibiotics for treatment of disease. There was also a general confusion among farmers surveyed since many farmers merely answered ‘did not know’, which may indicate they either do not want to or cannot answer the question.

The reasons for not using antibiotics was generally a matter of their fish or shrimp not becoming ill and, therefore, purchasing antibiotics was unnecessary, rather than a matter of preventing antibiotic residues from being present. A reason stated for not using antibiotics as a method of prophylaxis is that the heavy use of antibiotics slows growth, and, for snakehead farms, causes fish to become hump-backed or broken-backed, which would sell for a lower price. The other stated reason is the need to cut out antibiotics in order to meet the regulations to be able to sell their product for export, which brings in a higher income.

Without proper sanitation techniques, antibiotics can accumulate in the sediment at the bottom of aquaculture ponds for extended periods of time, creating constant pressure to bacterial populations that increase the risk of antibiotic resistance in bacterial pathogens found in fish or shrimp and water environment (Baquero et al. 2008; Pruden et al. 2013). Antibiotic resistance genes and bacteria have all been found in Vietnamese aquaculture products (Van et al. 2007, 2008). Furthermore, aquatic environments with higher antibiotic levels show more presence of antibiotic resistance genes, which are often situated on mobile genetic elements and can spread among various bacterial populations (Zhang et al. 2009). It is urgent to control and stimulate the proper use of antibiotics in aquaculture. Clear indications for antibiotic use and dosage should be established for Vietnamese aquafarmers. Upper limits for antibiotics in the environment need to be developed on a global level, with monitoring and appropriate action when levels are exceeded. Further studies that provide clear evidence of the link between inappropriate antibiotic use in aquaculture, and antibiotic residues and antibiotic resistance in bacterial pathogens, are needed to implement the appropriate control strategies.

## Electronic Supplementary Material

Below is the link to the electronic supplementary material.

Supplementary material 1 (DOCX 16 kb)
