# Supplementary material for: Monitoring Antibiotic Use and Residue in Freshwater Aquaculture for Domestic Use in Vietnam
Source: Ecohealth. 2015 Jan 6;12(3):480–9. doi: 10.1007/s10393-014-1006-z (PMC4623066; doi:10.1007/s10393-014-1006-z)
Supplement: Supplementary file 1 — Supplementary material 1 (DOCX 16 kb) [file 10393_2014_1006_MOESM1_ESM.docx]

**Table. Questionnaire to farmers about antibiotics use in aquaculture farms**

| **1) Antibiotic use in aquaculture farms**   1. Who usually administers drugs to the fish/shrimp? 2. Does that person have any training on the use of drugs? If yes, what kind of training? If no, how do you know how to use a drug? 3. Do you usually consult anybody for drug treatment? If yes, whom do you consult and how often? 4. Who usually buys the antibiotics used in your farm? 5. Do you keep records of aquaculture drugs used (name, dosage, route of administration, method for appliance, purchase price, quantity) on your farm? If yes, please show records. 6. Where do you buy the antibiotics used in your aquaculture farm? 7. Does the seller provide any information about the drug? If yes, what kind of information? 8. Do you continue drug treatment until harvest of aquatic products (fishes, shrimps)? If no, how many days before harvest do you stop using antibiotics? Why? 9. Where do you buy your feed? 10. Do you use feed additives? If yes, please list type of feed additives?   **2) Knowledge, perceptions and attitudes of farmers**   1. What do you do when you notice that your fish/shrimp are sick? 2. When do you decide to use an antibiotic? 3. Do you use any methods of sanitation and hygiene to prevent animal disease(s)? If yes, specify 4. What drugs do you use in case of diseased animals? (name of drug, dosage, route, duration for corresponding symptoms/signs) 5. Do you know of any government regulation(s) on the use of antibiotics? If yes, specify. 6. What type of customer you sell your fish/shrimp (market, other farm)? How do they influence your use of antibiotics? 7. What kind of relationship you have with other farms/drug or feed firms/agro-vet shop? How does this relationship influence your use of antibiotics? 8. Have you been visited by the local aquaculture inspector in the last 12 months? If yes, what was the purpose and result of visit? 9. Have you attended an aquaculture health-related seminar/field day/field demonstration in the last 12 months? 10. Have you ever attended a seminar/field day/field demonstration sponsored by a drug company? If yes, did they provide information on antibiotics? 11. Are you a member of an aquaculture-related community-based organization (CBO)? If yes, does the CBO provide aquaculture drug-related information? |
| --- |
